# Response of Common and Rare Beetle Species to Tree Species and Vertical Stratification in a Floodplain Forest

**DOI:** 10.3390/insects13020161

**Published:** 2022-02-03

**Authors:** Nora Haack, Paulo A. V. Borges, Annegret Grimm-Seyfarth, Martin Schlegel, Christian Wirth, Detlef Bernhard, Ingo Brunk, Klaus Henle, Henrique M. Pereira

**Affiliations:** 1 Biodiversity and Evolution, University of Leipzig, Talstraße 33, 04103 Leipzig, Germany; schlegel@rz.uni-leipzig.de; 2 German Centre for Integrative Biodiversity Research (iDiv) Halle-Jena-Leipzig, Deutscher Platz 5e, 04103 Leipzig, Germany; cwirth@uni-leipzig.de (C.W.); klaus.henle@ufz.de (K.H.); henrique.pereira@idiv.de (H.M.P.); 3 CE3C—Centre for Ecology, Evolution and Environmental Changes/Azorean Biodiversity Group, Universidade dos Açores, Rua Capitão João d’Ávlia, Angra do Heroísmo, 9700-042 Açores, Portugal; paulo.av.borges@uac.pt; 4 Department of Conservation Biology and Social-Ecological Systems, UFZ—Helmholtz Centre for Environmental Research, Permoserstraße 15, 04318 Leipzig, Germany; annegret.grimm@ufz.de; 5 Systematic Botany and Functional Biodiversity, University of Leipzig, Johannisallee 21, 04103 Leipzig, Germany; 6 Max-Planck Institute for Biogeochemistry, Hans-Knöll-Str. 10, 07745 Jena, Germany; 7 Molecular Evolution & Animal Systematics, University of Leipzig, Talstraße 33, 04103 Leipzig, Germany; bernhard@uni-leipzig.de; 8 Büro für Ökologische Gutachten, Cossebauder Strasse 3, 01157 Dresden, Germany; brunkin@web.de; 9 Institute of Biology, Martin Luther University Halle-Wittenberg, Am Kirchtor 1, 06108 Halle (Saale), Germany

**Keywords:** alpha diversity, beta diversity, abundance, community patterns, Coleoptera, floodplain forest

## Abstract

**Simple Summary:**

The vertical structure and the tree species in a forest have a strong influence on the communities of wood-inhabiting beetles. Little is known about how this influence varies between rare and common beetle species. We compared alpha and beta diversity patterns of common and rare species in the canopy of the Leipzig floodplain forest to assess their response to vertical structuring of the canopy and tree species. We assessed rarity firstly by using red list status and secondly based on the abundances of the beetle species. The understory displayed a significantly higher number of common species than the tree canopy. Conversely, the canopy harbored a higher number of rare species. The beetles’ beta diversity patterns were predominantly shaped by differences in species composition, not by differences in species richness. Vertical structure had a higher influence on the beetle communities than tree species. Both factors had a higher influence on common than on rare beetle species. Our results indicate that studies carried out in the understory alone do not allow drawing conclusions regarding the biodiversity in the forest canopy, and thus regarding the overall community structure of xylobiont beetles in the canopy.

**Abstract:**

Vertical stratification and host tree species are factors with a high influence on the structure of communities of xylobiont beetles. However, little is known about how this influence varies between common and rare species. Based on estimated species richness, we compared alpha and beta diversity patterns of common and rare species in the canopy of the Leipzig floodplain forest to assess their response to vertical stratification and tree species. We used two measures of rarity: threat level in red lists and abundance based on octaves. The understory displayed a significantly higher number of common species than the canopy strata. Conversely, the canopy strata harbored a higher number of rare species. Turnover was always dominant over richness differences in beta diversity partitions. Using Raup–Crick null models and non-metric multidimensional scaling, we found that the vertical strata accounted for 19% of the overall beta diversity of common species and for 15% of the overall beta diversity of rare species. The tree species accounted for 7% of the overall beta diversity of the common species and 3% of the beta diversity of the rare species. Our results indicate that studies carried out in the understory alone do not allow drawing conclusions regarding the biodiversity in the canopy strata, and thus regarding the overall community structure of xylobiont beetles in the canopy.

## 1. Introduction

In the current biodiversity crisis, it is crucial to understand the drivers of beta diversity at multiple scales as this provides insights about the mechanisms of beta diversity maintenance as well as about the way in which humans influence these mechanisms [1]. This knowledge is important for biodiversity conservation and management policies [2,3,4]. If the properties of a community like species richness and the spatial structure of dissimilarity are well-known, they can be the basis of conservation decision making. This reduces the need for detailed knowledge of the ecology of each species, which in some cases is almost impossible to obtain in a given time [5]. This is particularly helpful in species-rich and poorly studied taxa.

Forest ecosystems harbor a diversity of spatial components and dynamics [6,7,8], and with this a high and spatially structured diversity of habitats. Unlike most terrestrial ecosystems, forests do not only display horizontal patterns, e.g., by the position of tree species, but are also vertically layered. The effect of vertical stratification has been examined in various animal taxa such as bats, e.g., [9,10], birds, e.g., [11,12], bees, e.g., [13,14], spiders, e.g., [15,16,17], and beetles, e.g., [15,18,19,20,21]. The results, however, are diverse even within groups, likely dependent on the type of forest, the management of the forest, and the definition of the vertical strata [22,23].

Among the temperate forest ecosystems, floodplain forests harbor an outstanding richness of structures and biodiversity [24,25,26]. As in many floodplain forests, the fluvial dynamics in the Leipzig floodplain forest are disturbed by human interventions. It remains, however, a near-natural deciduous forest [27,28] with a high species richness of various arthropods such as spiders [29] and xylobiont beetles [21].

Insects, and beetles in particular, are more species-rich than any other animal taxon. Due to their taxonomic and functional diversity, they are of ecological importance, but they also pose special challenges for the assessment of diversity [30]. In forest ecosystems, many insect species are directly or indirectly dependent on living or dead wood [31]. Among these, the most diverse are xylobiont beetles. They rely on very fine-scale habitat features such as the decay stage, position, and tree species origin of dead wood as well as the composition of wood-decomposing fungal communities [32]. Therefore, a high beta diversity can occur between microhabitats, and ecological studies have to address variation in these determining features at small scales. Although beetles are flight-active and have been proven to be able to disperse over many kilometers in flight mill experiments [33,34,35,36] and in genetic studies [37,38], field experiments revealed that they tend to roam close to suitable habitats [39].

The tree species composition in a forest stand should have an impact on the community of xylobiont beetles, which often have specialized ways of feeding and foraging and include many oligo- and even mono-phageous species [32]. Generally, the host preferences of saproxylic species are not well known, as feeding experiments would be time- and cost-intensive [40]. However, there are studies indicating that host tree availability should indeed show an effect on the communities of xylobiont beetles. Some families such as bark beetles are known to be host-specific [41]. On the other hand, two genera of trees common in the Leipzig floodplain forest, *Tilia* and *Fraxinus*, are rather avoided by xylobiont beetles [40]. Therefore, we expect an influence of tree species on the communities of xylobiont beetles.

Richness patterns for rare and common species have often been found to differ [42,43,44,45]. It is also often assumed that common species are less sensitive to environmental changes, and conservation strategies mostly focus on rare species. Nevertheless, some studies show that environmental factors predict the richness patterns of common species better than they do for rare species [42,43,46]. The concept of rarity itself is sometimes complex. For red listing, IUCN uses several criteria based on the occurrence and abundance of species and extinction risk. Rabinowitz [47] described seven forms of rarity, the most drastic form of rarity being a species with low abundance, restricted range size, and habitat specificity. Gaston [48] proposed the 25% rule that can be applied in several forms (e.g., the 25% most rare species in the community in terms of abundance or occurrence; or all species with less than 25% of the maximum value of abundance or occurrence). Currently, most studies use a relative measure of rarity using one or more criteria (see for a summary Kondratyeva et al. [49]).

In the present study, we assess the effects of vertical stratification on the alpha and beta diversity of xylobiont beetles within the canopy and how they differ between the canopy and the near-ground stratum. We identify the effect of tree species on the beetle communities and compare it to the effect of stratification. Furthermore, we compare these effects among rare versus common beetle species. These analyses are a step towards a better understanding of how the communities of xylobiont beetles are structured and, therefore, they can potentially aid the conservation of forest ecosystems.

## 2. Material and Methods

### 2.1. Sampling Location and Design

Sampling was conducted in the Burgaue nature reserve in the Leipzig floodplain forest, a near-natural deciduous forest that largely consists of six main tree species *Quercus robur*, *Fraxinus excelsior*, *Tilia cordata*, *Acer pseudoplatanus*, *Ulmus laevis,* and *Carpinus betulus*. This forest is in the focus of biodiversity studies within the Leipzig Canopy Crane (LAK) and the Lebendige Luppe projects [50,51]. Canopy sampling was facilitated by means of the Leipzig Canopy Crane, a revolving tower crane (Liebherr 71 EC), 40 m high and spanning an area of 16.500 m^2^. The main autochthonous tree species, *Q. robur*, *T. cordata,* and *F. excelsior*, were selected for sampling beetles using window traps. Thirty traps were installed in two strata (20 and 25 m height) within the three key tree species. Those height differences were chosen due to the actual heights of the canopies of the sampled trees. Given the extreme differences in light and microclimate that exist on this scale, we are certain that they provide very different microhabitats. For *T. cordata* and *F. excelsior,* five trees were sampled, each with one trap in the canopy and one in the middle stratum. For *Q. robur*, only four individual trees were accessible. However, on the largest oak we installed two window traps per stratum in the opposite direction, yielding a sampling distance almost equal to those between different trees. The distance between individual trees differed between approximately 10 and 120 m as trees had to be chosen based on the accessibility by the crane. Additionally, a total of eight understory traps were installed at a height of two meters. The traps used were omnidirectional window flight intercept traps [21,52] that consisted of an upper sampling unit, a lower sampling unit, and two inter-crossed plexiglass panels. The upper and lower sampling units each consisted of a sampling container filled with diethylene glycol in the upper and lower canopy stratum. We chose diethylene glycol here as the higher temperatures and exposure to sunlight in the canopy promote evaporation, and ethylene glycol is more resistant to evaporation than ethanol. As diethylene glycol poses a potential threat to people, in the near-ground stratum we had to use ethanol instead. It needs to be considered, however, that this necessary decision could have biased the results due to ethanol being a potential attractant, especially for bark beetles [53]. Sampling was performed biweekly, 13 times from 29 March 2017 to 29 September 2017. This sampling period covered the main activity period for arthropods in our study area. The biweekly intervals of collection ensured that evaporation of sampling liquids and damage of the collected specimens were minimized.

### 2.2. Species Determination and Composition

All coleopterans were determined at the species level using mainly Freude, Harde, Lohse (1964–1983), and supplements. In total, 442 species were identified; 237 species were excluded from further analyses as they were not xylobiont beetles; 205 species of xylobiont beetles were found and selected for further analyses. Our dataset is stored in the Dryad data portal, where it will be accessible to the wider public. The DOI is *XX—to be inserted upon acceptance*. Voucher specimens are deposited at the zoological collection of the University of Leipzig.

### 2.3. Rarity Definitions

We assessed rarity using two different approaches; this minimized potential biases that may be associated with each particular method. In the first approach, we defined the rarity of species independently of our data by using the red list for beetles of Germany [54]. All rare species in the red list categories 1–3 (Appendix A.1) were considered as rare for our analyses. The red list, though a popular instrument in conservation, comes with some disadvantages. The available red list was not specific for the study region or ecosystem, but rather represented an average value of rarity for Germany as a whole. In addition, it did not include the categories ‘data deficient’ or ‘least concern’, and all species that did not fall in the above-mentioned categories were assumed to be common species. Therefore, we also assessed rarity using an approach that built on abundances within our own data set.

We used the concept of octaves developed by Preston [55,56], using modified log_2_ classes, and following method 3 as proposed by Gray et al. [56], which was regarded as the most appropriate binning method in their study. In this method, all species with only one individual caught are assigned to bin 1, all species with 2–3 individuals to bin 2, all species with 4–7 individuals to bin 3, all species with 8–15 individuals to bin 4, defining the interval on a log_2_ scale. The octaves were calculated using the R package gambin [57]. We considered species in octaves 1–6 (species with up to 63 individuals) to be the rare species. This included 183 xylobiont species (89% of all xylobiont species). We set this threshold as this is the lowest octave that still provides reliable estimates for beta diversity when using the SpadeR package. The results for the rarity definition according to the octaves are shown in Figure A6, Figure A7, Figure A8, Figure A9, Figure A10, Figure A11, Figure A12, Figure A13 and Figure A14 in Appendix A.2.

### 2.4. Statistical Analyses

#### 2.4.1. Alpha Diversity

Alpha diversity was assessed using the R package iNEXT [58,59] to calculate non-asymptotic estimates of species richness from rarefaction and extrapolation sampling curves and estimates of sampling coverage. We extrapolated each subset (stratum or tree species) to up to 30 traps, which was twice our highest actual number of traps used (8 traps were used in the understory, 15 in each of the canopy strata, and 10 in each of the tree species).

#### 2.4.2. Beta Diversity

##### Trapwise Community Dissimilarity

As an earlier study [21] in our study area showed that the raw data do not yield a reliable result on shared species between strata or tree species, we used the SimilarityMult-function of the R package SpadeR [60] to calculate multiplicative beta diversity estimates with 200 bootstraps. We set q = 1 to yield the N-community Horn similarity measure as a pairwise matrix [61]. To obtain dissimilarity, we subtracted each value of this matrix from 1. The resulting dissimilarity matrix was used as the basis for all further beta diversity analyses.

We calculated two dissimilarity matrices: one for the three strata, including all traps, and another for different tree species, excluding the traps from the understory, which could not be annotated to a particular tree species. Abundance-based estimates were chosen as we assumed they might be less sensitive to sample size than presence–absence measures and our sample sizes differed between the near-ground stratum and the canopy strata in our study.

##### Replacement versus Richness Differences

The contribution of replacement and richness differences to the overall beta diversity was studied using the beta function in the R package ‘BAT’ [62]. Beta diversity was partitioned using the methodology developed by Carvalho [63]. First analyses showed that replacement accounted more for beta diversity than differences in species richness. Therefore, we decided against the beta diversity partitioning methodology proposed by Baselga [64] as it has been shown to overestimate replacement [63,65]. The total beta diversity and the contribution of replacement and richness differences were calculated once for all samples together and once per studied tree species and stratum.

##### Assessment of Stochastic and Deterministic Processes

To assess to what extent trapwise dissimilarity is caused by deterministic and stochastic processes, Raup–Crick null models were created. These models randomly reshuffle species identities among the samples, generating a null expectation under the assumption that beta diversity is randomly distributed. The models were calculated in R using the ‘raup-crick’ function provided by Chase et al. [66]. It is important to notice that the functions treated our abundance data as presence–absence data. Null models were calculated separately for strata and tree species, each with 1000 replications.

Boxplots were then used to visualize the mean trapwise dissimilarity when comparing different strata and tree species. The analyses were carried out for common and for rare beetle species separately. Patterns of variation in species composition between the traps were visualized through non-metric multidimensional scaling (NMDS) ordinations. The NMDS was plotted separately for common and rare beetle species.

The influence of stratum and sampled tree species on the variation in species composition was then quantified through a PERMANOVA with the R function ‘adonis.’ A matrix regression with the dissimilarity matrix as the dependent variable and an environmental matrix as the independent variable was performed. The analyses were run separately with either tree species or stratum as the independent variable. NMDS and PERMANOVA analyses were carried out in ‘vegan.’ All analyses were carried out for common and for rare beetle species separately using R version 4.1.0 [67].

## 3. Results

### 3.1. Alpha Diversity of Xylobiont Beetle Communities

**Figure 1:**
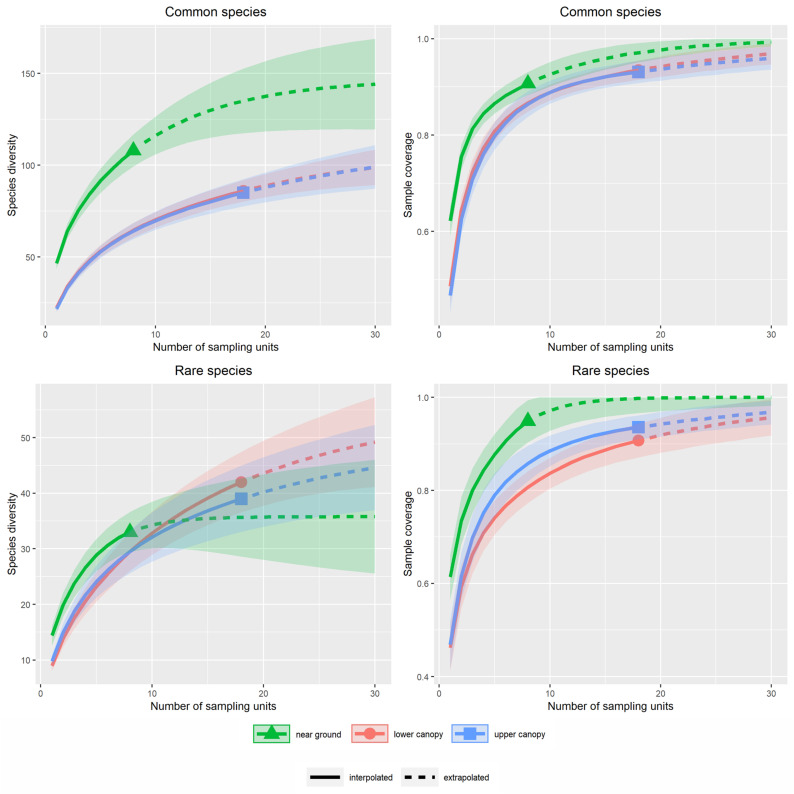
Comparison of species richness and sample coverage among the studied strata for rare and common beetle species, according to the red list definitions. The left column shows the estimated species richness against the number of traps per stratum. The right column shows the sample coverage against the number of traps per stratum. The bands mark the 95% confidence interval.

Application of the red list criterion of commonness and rarity revealed a considerably higher richness for the common species in the understory than in the canopy strata. For the rare beetle species, we found a higher richness in the canopy strata than in the understory, which, however, was not significant (Figure 1). When defining rarity according to the octaves, a different pattern was found. Here, the species richness of the rare and common beetle species was higher in the understory, although for the common beetle species the difference was not significant (Figure A6).

For the common beetle species, sample coverage was higher for the canopy strata than for the understory. For none of the strata, a complete saturation was reached at our actual number of samples, but for all strata, a flattening of the curve was visible. With the extrapolated 30 sampling units, the curves were close to saturation. Sample coverage was higher for the understory than for the canopy strata (Figure 1).

**Figure 2:**
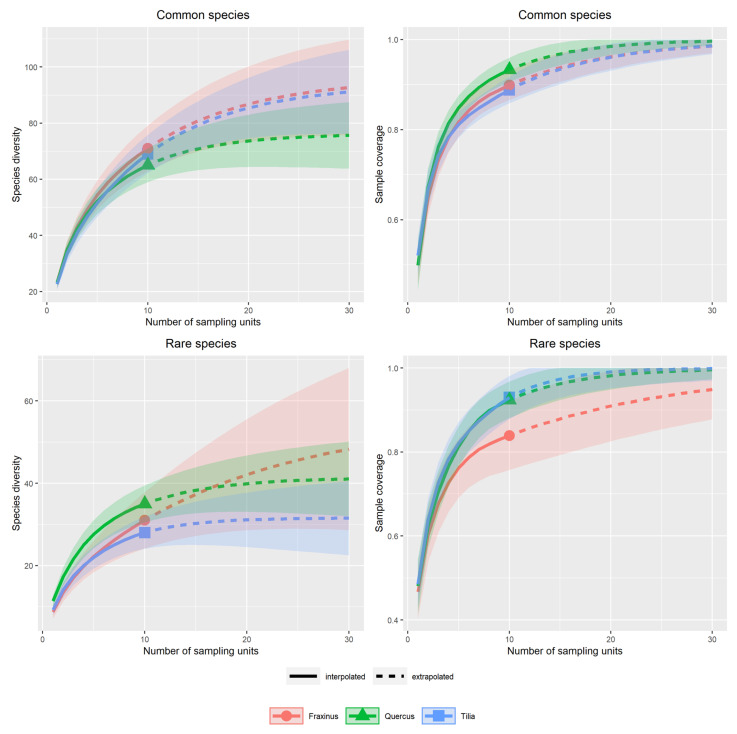
Comparison of species richness and sample coverage among the studied tree species for rare and common beetle species, according to the red list definitions. The left column shows the estimated species richness against the number of traps per tree species. The right column shows the sample coverage against the number of traps per tree species. The bands mark the 95% confidence interval.

No significant difference in species richness was found among the tree species, neither for the rare nor the common beetle species. This was true for both definitions of rarity (Figure 2, Figure A7).

For the common beetle species, full sample coverage was not reached by our number of sampling units in any tree species. With the extrapolated 30 sampling units, the curves were close to saturation, more so for *Q. robur* than for the other tree species. For the rare beetle species, a much lower sample coverage was reached in *F. excelsior* than in the other two tree species (Figure 2).

### 3.2. Beta Diversity of Xylobiont Beetle Communities

#### Replacement versus Richness Differences

Total beta diversity, measured as Jaccard dissimilarity, averaged 0.73 between a pair of sites for common species and 0.76 between a pair of sites for rare species. On average, replacement accounted for 0.46 for common and 0.52 for rare species. It was dominant over richness differences, which, on average, accounted for 0.27 for common species and 0.24 for rare species. This pattern was shown throughout all studied strata and tree species for common and rare beetle species (Appendix A.1.2). Using the octaves as rarity categories yielded similar results (Appendix A.2.2).

### 3.3. Environmental Influences

#### 3.3.1. Assessment of Stochastic and Deterministic Processes of Trapwise Dissimilarity Using Raup–Crick Models

**Figure A2:**
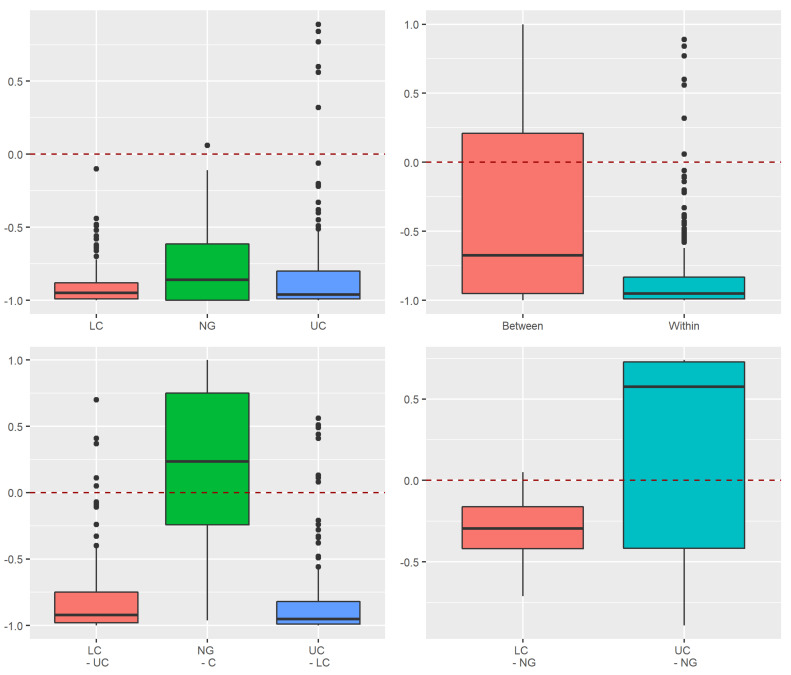
Deviation of the beta diversity from the diversity expected by the null hypothesis per stratum for the common beetle species. NG—near-ground stratum, LC—lower canopy, UC—upper canopy. Comparisons within a stratum are comparisons of two traps of the same stratum. Comparisons between strata are comparisons of two traps from two different strata.

**Figure A3:**
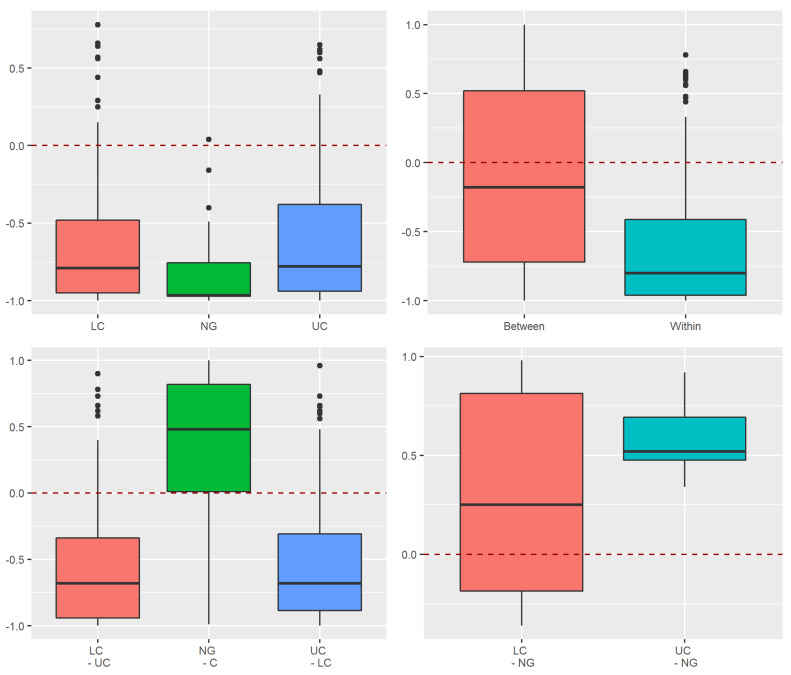
Deviation of the beta diversity from the diversity expected by the null hypothesis per stratum for the rare beetle species. NG—near-ground stratum, LC—lower canopy, UC—upper canopy. Comparisons within a stratum are comparisons of two traps of the same stratum. Comparisons between strata are comparisons of two traps from two different strata.

For the common beetle species, the observed dissimilarity between two traps within the strata was lower than expected under the null model (Figure A2). However, when comparing a trap from the near-ground stratum to a trap from any of the canopy strata, the observed dissimilarity was higher than expected based on any of the simulations. The observed dissimilarity between the upper canopy and the near-ground stratum was higher than between the lower canopy and the near-ground stratum (Figure A2). For the rare beetle species, the pattern was very similar. In general, heterogeneity within groups was higher for the rare species (Figure A3).

**Figure A4:**
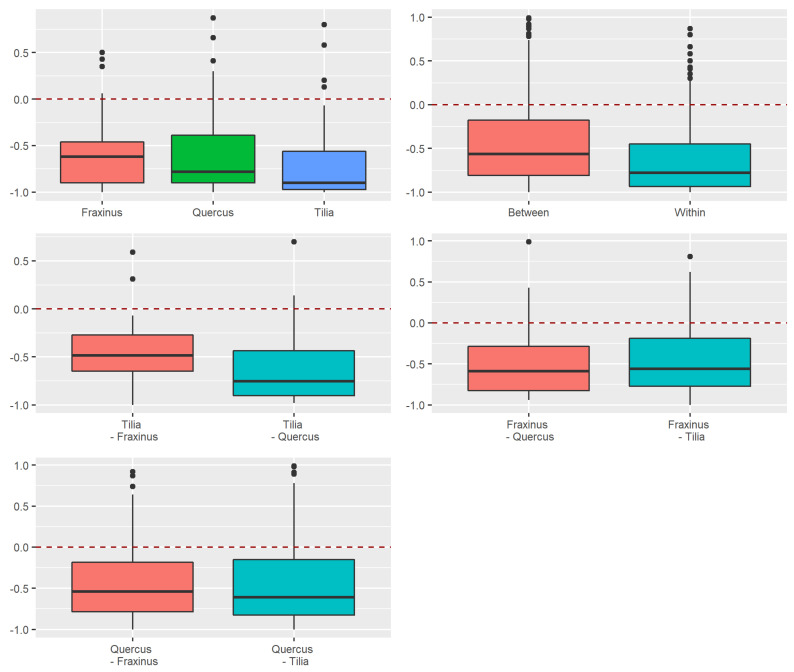
Deviation of the beta diversity from the diversity expected by the null hypothesis per tree species for the common beetle species. Comparisons within a tree species are comparisons of two traps of the same tree species. Comparisons between tree species are comparisons of two traps from two different tree species.

**Figure A5:**
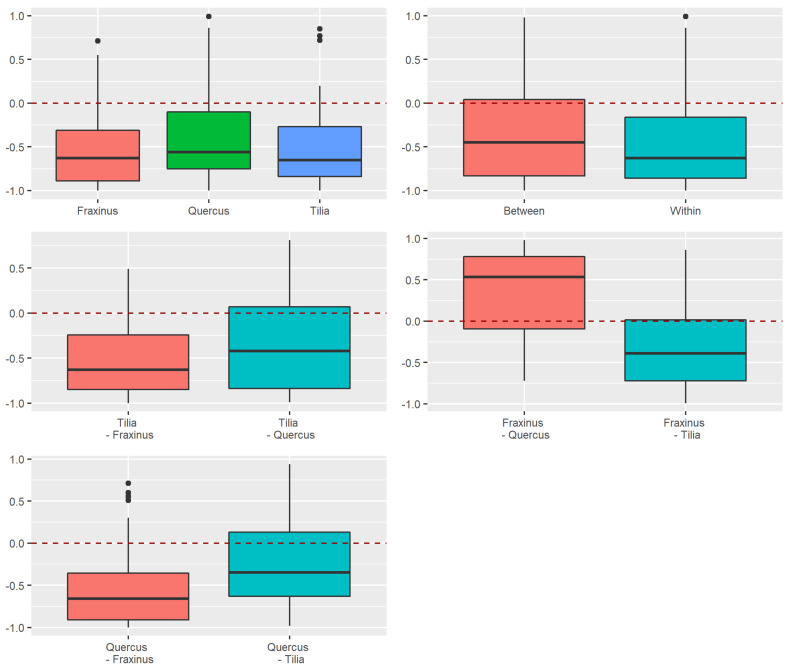
Deviation of the beta diversity from the diversity expected by the null hypothesis per tree species for the rare beetle species. Comparisons within a tree species are comparisons of two traps of the same tree species. Comparisons between tree species are comparisons of two traps from two different tree species.

**Figure A6:**
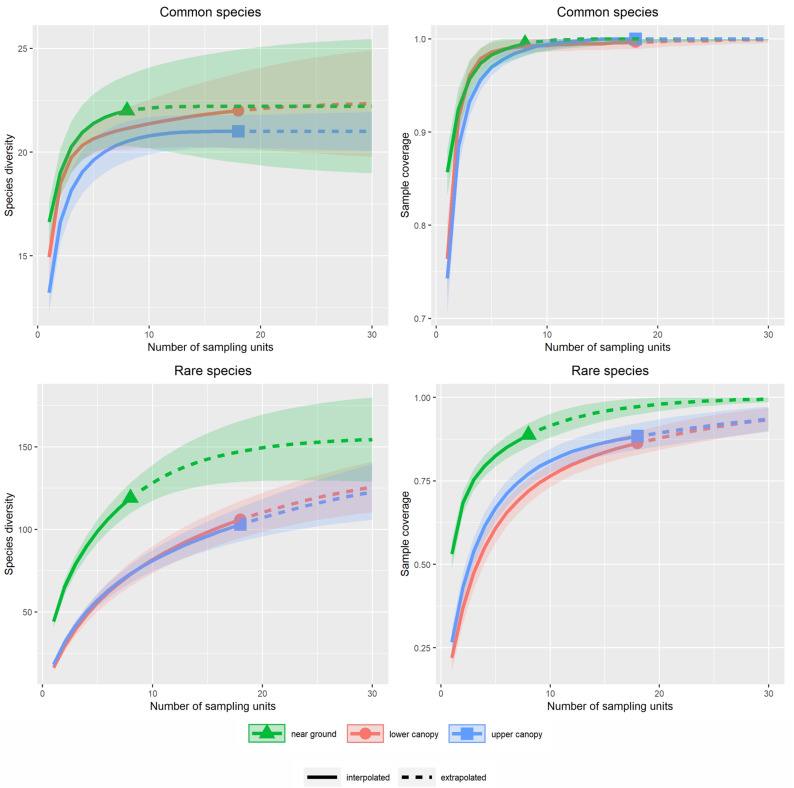
Comparison of species richness and sample coverage among the studied strata for rare and common beetle species, rarity classification according to the octaves.

**Figure A7:**
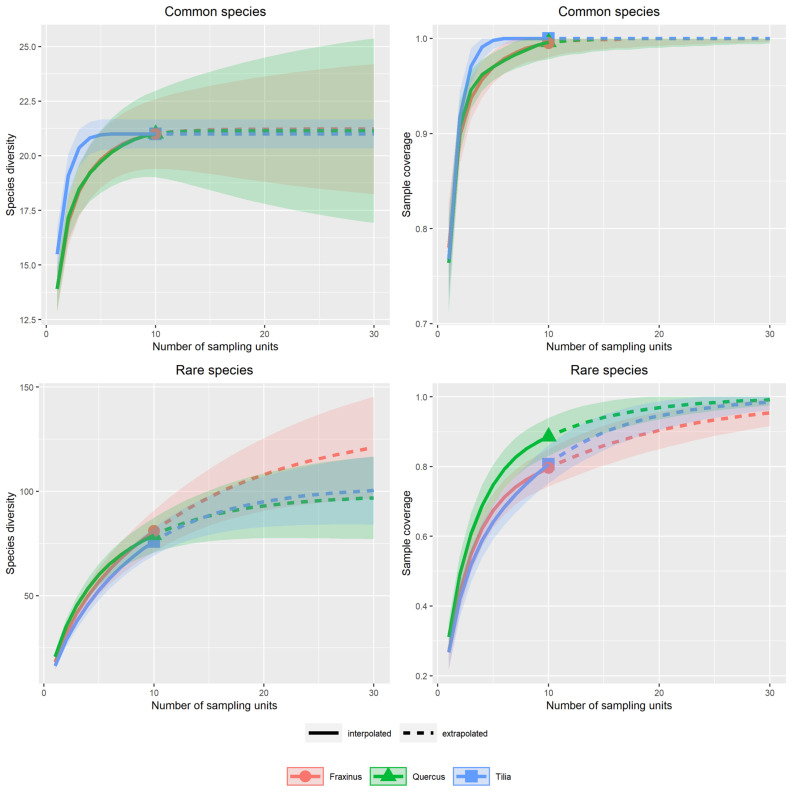
Comparison of species richness and sample coverage among the studied tree species for rare and common beetle species, rarity classification according to the octaves.

**Figure A8:**
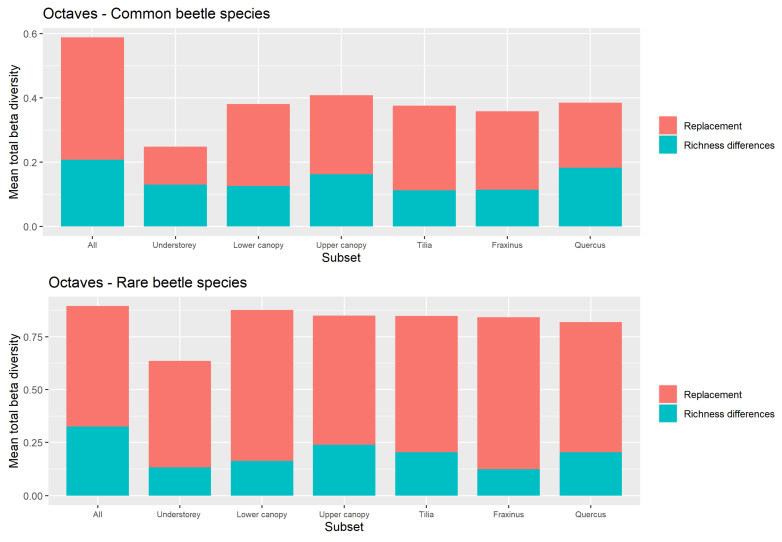
The contribution of replacement and richness differences to the total beta diversity for all studied strata and tree species, rarity classification according to the octaves.

**Figure A9:**
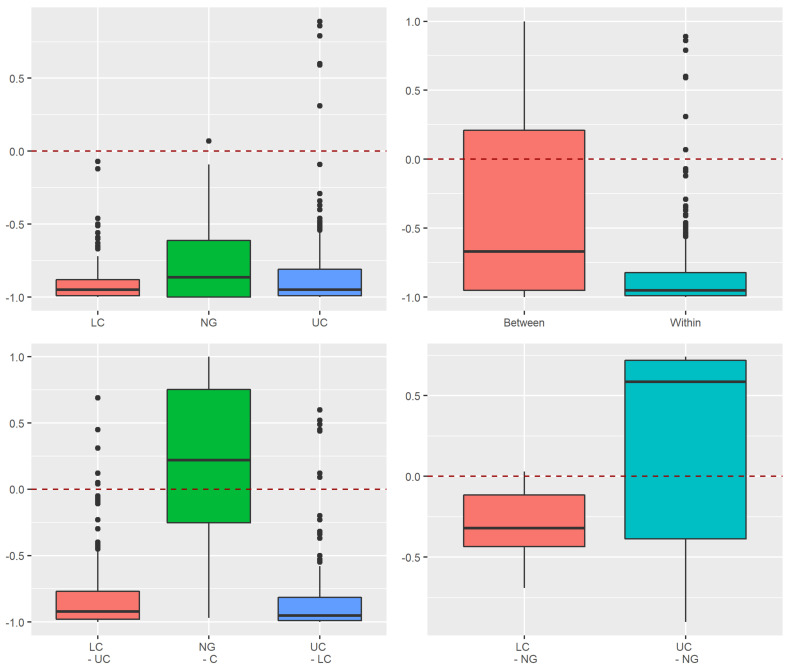
Deviation of the beta diversity from the diversity expected by the null hypothesis per stratum for the common beetle species, rarity classification according to the octaves. NG—near-ground stratum, LC—lower canopy, UC—upper canopy. Comparisons within a stratum are comparisons of two traps of the same stratum. Comparisons between strata are comparisons of two traps from two different strata.

**Figure A10:**
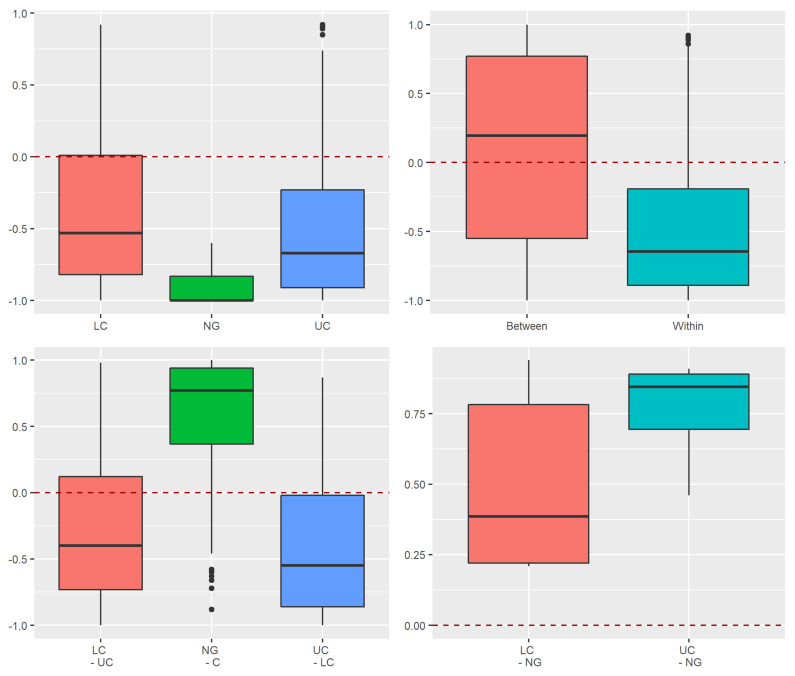
Deviation of the beta diversity from the diversity expected by the null hypothesis per stratum for the rare beetle species, rarity classification according to the octaves. NG—near-ground stratum, LC—lower canopy, UC—upper canopy. Comparisons within a stratum are comparisons of two traps of the same stratum. Comparisons between strata are comparisons of two traps from two different strata.

**Figure A11:**
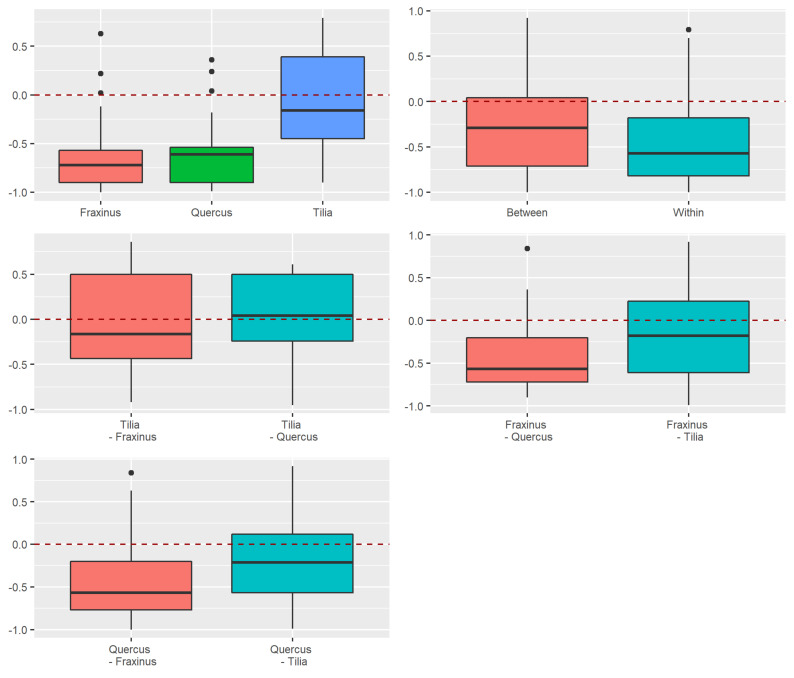
Deviation of the beta diversity from the diversity expected by the null hypothesis per tree species for the common beetle species, rarity classification according to the octaves. Comparisons within a tree species are comparisons of two traps of the same tree species. Comparisons between tree species are comparisons of two traps from two different tree species.

**Figure A12:**
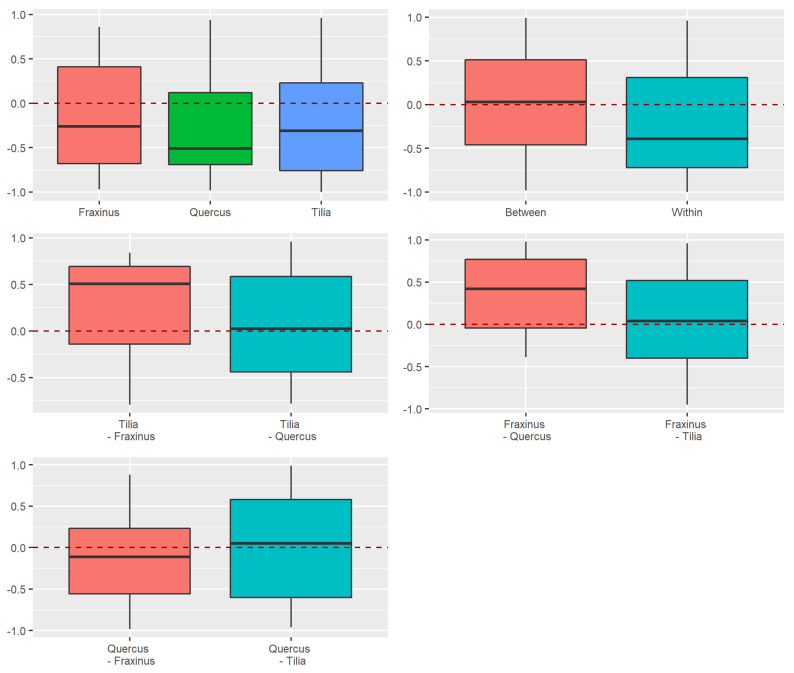
Deviation of the beta diversity from the diversity expected by the null hypothesis per tree species for the rare beetle species, rarity classification according to the octaves. Comparisons within a tree species are comparisons of two traps of the same tree species. Comparisons between tree species are comparisons of two traps from two different tree species.

For the common beetle species, the observed dissimilarity was lower than expected by the null model, within and between the studied tree species. The variance in dissimilarity was higher when comparing two traps from two different tree species than when comparing two traps from the same tree species. When comparing the tree species pairwise, all showed a dissimilarity lower than expected by the null model (Figure A4). For the rare beetle species, the dissimilarities between tree species, however, were overall higher than expected by the null model. In the pairwise comparisons, *F. excelsior* and *T. cordata* showed a dissimilarity that was considerably higher than expected by the null model (Figure A5). Due to the pairwise analyses, pairings of two tree species are treated differently depending on whether the first or the second trap comes from any certain tree species. The differences between these pairings (e.g., *T. cordata—Q. robur* versus *Q. robur—T. cordata*) are to be regarded as artifacts and were not significant in any of the combinations. The patterns were similar when rarity was assessed based on the octave distribution (Figure A9, Figure A10, Figure A11 and Figure A12).

#### 3.3.2. Dissimilarity between Strata and Tree Species

**Figure 3:**
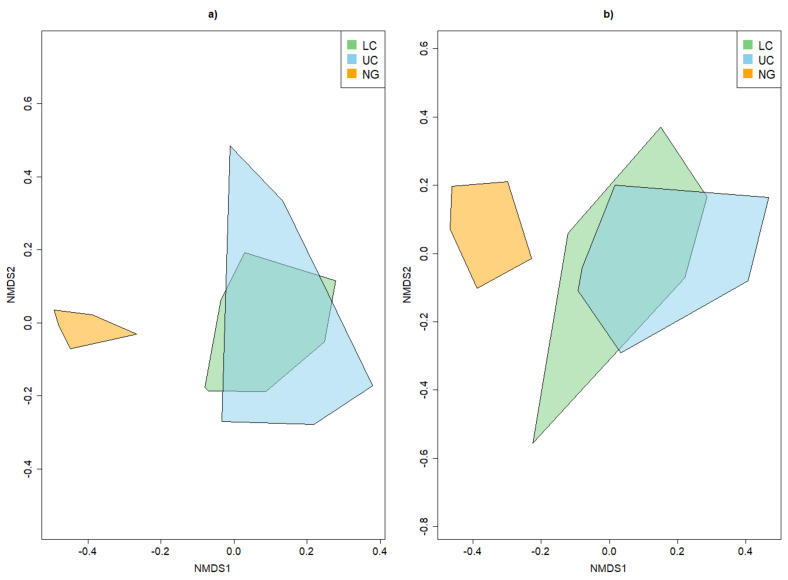
NMDS plot demonstrating the influence of strata on the overall beta diversity for common beetle species (**a**) and only the rare beetle species (**b**), according to the red list categories. NG—near-ground stratum, LC—lower canopy, UC—upper canopy.

**Figure A13:**
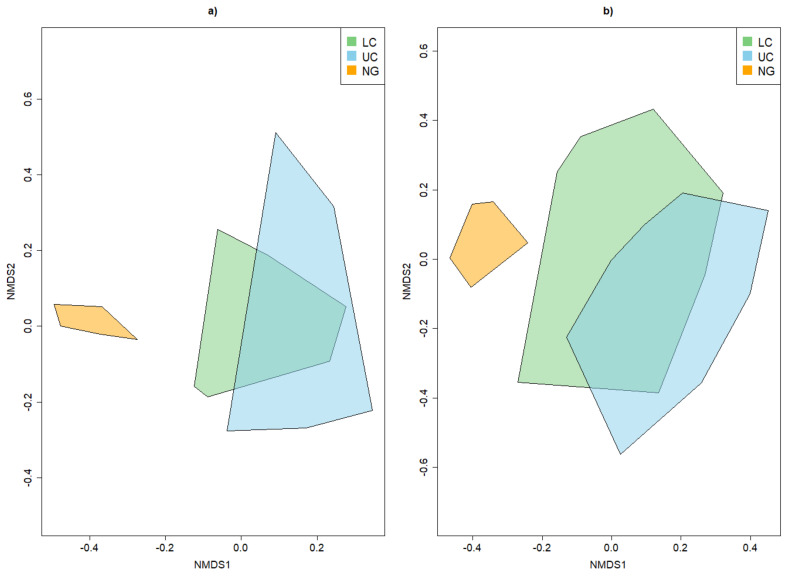
NMDS plot demonstrating the influence of strata on the overall beta diversity for the common beetle species (**a**) and the rare beetle species (**b**), rarity classification according to the octaves. NG—near-ground stratum, LC—lower canopy, UC—upper canopy.

**Figure A14:**
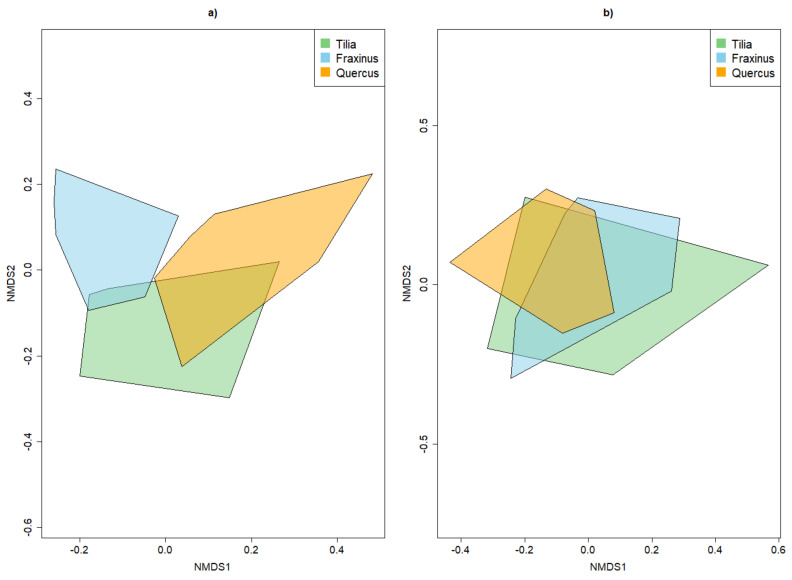
NMDS plot demonstrating the influence of tree species on the overall beta diversity for the common beetle species (**a**) and the rare beetle species (**b**), rarity classification according to the octaves.

The NMDS plots for the common beetle species showed an overlap of the two canopy strata, while the near-ground stratum showed no overlap with any canopy stratum (Figure 3, Figure A13). Furthermore, the beetle community in the understory spanned the smallest area in ordination space. This pattern was found in the rare and common beetle species alike (Figure 3, Figure A13).

**Figure 4:**
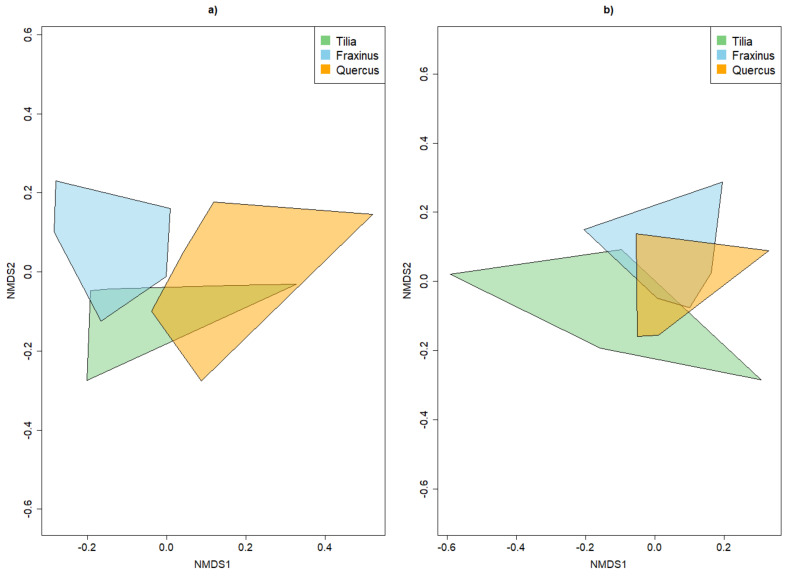
NMDS plot demonstrating the influence of tree species on the overall beta diversity for common beetle species (**a**) and only the rare beetle species (**b**), according to the red list categories.

For common species, there was an overlap between *T. cordata* with the two other tree species, while *Q. robur* spanned the largest area in ordination space (Figure 4). If only the rare beetle species were considered, all tree species overlapped; this was more pronounced between *Q. robur* and *T. cordata* than between *F. excelsior* and *T. cordata*. *T. cordata* spanned the largest area in ordination space (Figure 4).

The same patterns were obtained when rarity was classified with the octaves approach (Appendix A.2.4). Only the overlap between all tree species for the rare beetle species was larger compared to the red list criterion. PERMANOVA further supported the finding that strata had a higher influence on species composition than tree species and that the species composition was better explained for the common than the rare beetle species by strata and tree species alike, as shown by the F statistics (Table 1).

## 4. Discussion

### 4.1. Alpha and Beta Diversity between the Strata

The communities of the understory and the canopy differed significantly in alpha and beta diversity for both rare and common beetle species. We found that species richness was higher in the understory than in the canopy strata for the common species but lower for the rare beetle species when using the red list definition of rarity. When using the octave definition of rarity, species richness was higher in the understory for both groups. This discrepancy might be explained by the red list likely being compiled mostly from the easier-to-access understory. Consequently, species with a preference for canopy habitats may be more often classified artificially as rare species in the red lists. Common species were found to be more species-rich in the understory with both rarity assessments, although the difference was more distinct with the red list classification. This is likely due to a higher heterogeneity of the understory habitat, which harbors not only seedlings of the canopy tree species, but also the vast majority of herbaceous plants in temperate forests [68]. Earlier studies also explained higher insect diversity in the understory of temperate forests with the accumulation of detritus at the forest floor and the high availability of edible vegetation in the shrub layer [69].

It is widely assumed that temperate forests are less stratified than tropical forests due to fewer structural differences and seasonal changes in climatic conditions, which force many species to migrate between strata [22]. Nevertheless, our study showed that the communities of xylobiont beetles in the canopy were distinct from the communities in the understory. This is probably due to the structure of the Leipzig floodplain forest with its comparatively high canopy of many old trees [70] supporting an elevated vertical heterogeneity, which makes microhabitat segregation between arthropod communities more likely [22,71]. Furthermore, the high age and large size of oak and ash trees of the Leipzig floodplain forest in particular promote a high diversity of structures such as tree cavities, above-ground soil, fungi, and epiphytes such as lichen and moss. They are important microhabitats for xylobiont arthropods and are not evenly distributed in the canopy [22]. Therefore, they also increase vertical beta diversity. It should also be noted that due to a lack of regular flooding events since the mid-20th century, a second layer of shade-tolerant but not flooding-tolerant Sycamore has developed that currently creates very dark and moist conditions in the understory [28]. This second canopy possibly increases the microclimatic gradient between the understory and the canopy, thus potentially amplifying differences in their beetle communities. It needs to be considered that a bias might have been introduced by the use of different sampling fluids. This could contribute to, but not fully explain, differences in alpha and beta diversity between canopy strata and the understory. As studies using the same sampling fluid over strata found similar differences between the understory and the canopy [20], we can assume that these differences are not the result of a difference in sampling fluids alone.

Our analyses emphasized that results from the understory cannot be generalized to the canopy. This is not surprising when considering the ecological specializations of xylobiont beetles. Woodboring beetles are known to be more attracted to standing dead wood while many fungivorous species prefer fallen trees and are therefore more likely to occur in the understory [22]. Furthermore, xylobiont beetle species that target branches and snags are more likely to be present in the canopy than in the understory [22]. Therefore, to gain a more complete overview of forest diversity, we suggest that sampling in forest habitats should be extended to the canopy wherever possible.

### 4.2. Alpha and Beta Diversity between Tree Species

The alpha diversity of beetle species did not differ significantly between tree species. Moreover, overall, beta diversity between tree species was more similar than expected from the random distribution in the null models and showed an overlap in the NMDS plots for almost all combinations (Figure 3). Oak is largely considered to be of special importance for the biodiversity of xylobiont insects [72,73], which was not confirmed by our results. *Q. robur*, historically dominant in the floodplain forest [70], did not show a higher beetle species richness than *T. cordata* and *F. excelsior*. This pattern was similar for both definitions of rarity that we tested. For *F. excelsior*, this finding may be explained by the increased dead wood production in the crown in *F. excelsior* that stems from infection with ash dieback, resulting in an enlarged quantity of potential microhabitats. The lack of significant differences between the tree species might also be partly due to our sampling scheme. It is plausible that window flight traps provide less information about host specificity than sampling methods such as stem collectors or breeding larvae from sampled wood. In fact, studies that bred larvae directly from logs found higher values of host specificity [40]. Nevertheless, a range of studies with different sampling techniques has come to similar results, supporting a lower tree species specificity [74,75,76]. It is likely for many xylobiont beetle species that the importance of the tree species decreases with ongoing wood decomposition [40,77]. Generally, it needs to be considered that tree species and vertical stratification are not independent of each other, as plant diversity also has an effect on the vertical stratification of forest ecosystems [13,14]. Vertical stratification often comes from a high diversity of shrubs and trees, which, due to different habitat specializations such as shade tolerance, contribute to structuring canopy layers. Therefore, a high diversity of tree species is needed to form full vertical gradients in a forest.

### 4.3. Beta Diversity Partitioning into Richness Differences and Replacement

Replacement was always dominant over richness differences. This suggests that the beta diversity of beetles in the Leipzig floodplain forest is most likely a result of an environmental gradient or competition, emphasizing the role of deterministic, niche-based processes in shaping the communities of xylobiont beetles in the Leipzig floodplain forest. Similar patterns were found in other temperate and tropical forests [78]. The niche availability seems to be similar over strata and tree species. This pattern is important for conservation efforts. In an ecosystem where replacement plays a large role, the environmental gradients have to be maintained to protect biodiversity [1]. We therefore recommend that in the case of the Leipzig floodplain forest, not only should the diversity of trees be targeted by conservation measures but also the vertical diversity of the canopy.

## 5. Conclusions

Vertical stratification and tree species affected the diversity of common species more strongly than rare species. The effect of tree species and stratification on beta diversity was stronger for common species. This result might partly be linked to species with strong preferences for common combinations of environmental factors [79]. If some of the common species are indeed specialized on stratum- or tree species-specific niches that are however common in the Leipzig floodplain forest, they will strongly differentiate between strata and trees because, as common species’ sample sizes are much larger, power for differences is also larger. It seems plausible that rare species are specialists, but they specialize in factors that are rare in the floodplain forest. Therefore, rare species come into the floodplain forest more randomly from the outside. Thus, it might be difficult to find specific factors common to all rare species that explain their richness patterns. We found substantial differences in alpha and beta diversity between the understory and the canopy strata, whereas these differences were less pronounced between tree species. We strongly suggest always taking the canopy region into account for an assessment of the diversity of xylobiont beetles. Further research on the host specificity of xylobiont beetles in the Leipzig floodplain forest using more tree-specific sampling techniques seems necessary to fully grasp the importance of tree species diversity for the communities of xylobiont beetles. We consider that our results might also apply to other arthropod species communities.

## Figures and Tables

**Table 1:** PERMANOVA quantification of the impact of different environmental sources of variation on the dissimilarities between traps.

Rarity	Factor	Degree of Freedom	Sum of Squares	R-Squared	F Statistics	*p*-Value
Common	Stratum	2	2.40	0.48	19.10	0.001
	Residual	41	2.58	0.52		
	Total	43	4.99	1.00		
Rare	Stratum	2	2.32	0.42	15.07	0.001
	Residual	41	3.16	0.58		
	Total	43	5.49	1.00		
Common	TreeSp	2	0.72	0.36	7.51	0.001
	Residual	27	1.30	0.64		
	Total	29	2.02	1.00		
Rare	TreeSp	2	0.51	0.19	3.25	0.003
	Residual	27	2.13	0.81		
	Total	29	3.00	1.00		

**Figure A1:**
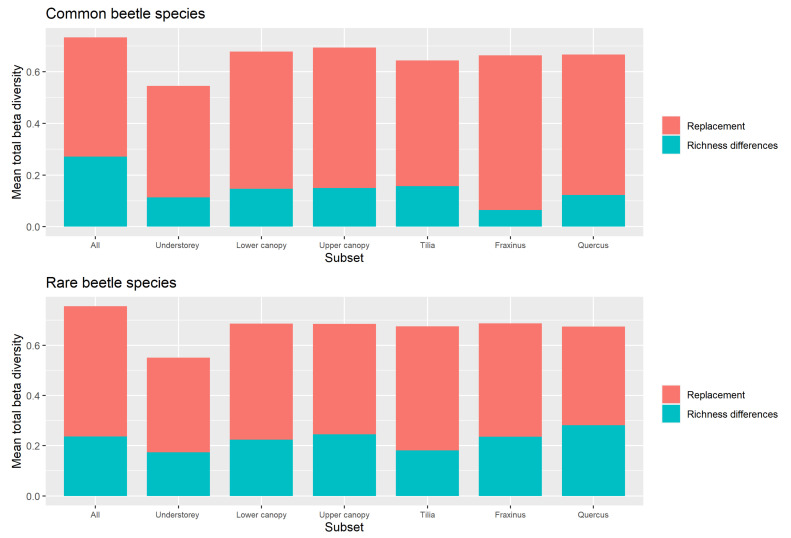
The contribution of replacement and richness differences to the total beta diversity for all studied strata and tree species.

## Data Availability

The data that support the findings of this study are openly available in the OSF data repository at DOI 10.17605/OSF.IO/9DHZW, https://osf.io/9dhzw/ (accessed on 16 November 2021).

## Appendix A

### Appendix A.1. Supplementary Graphs for the Use of Red Lists for the Definition of Rarity

#### Appendix A.1.1. Categories of the Red List Used for the Present Analyses

**Table A1:** The red list categories used in the red list of beetles (Geiser, 1998).

Category	Significance
0	Extinct
1	Threatened with extinction
2	Highly endangered
3	Endangered
